# Effects of Taekwondo training on physical fitness factors in Korean elementary students: A systematic review and meta-analysis

**DOI:** 10.20463/jenb.2019.0006

**Published:** 2019-03-31

**Authors:** Sang-Seok Nam, Kiwon Lim

**Affiliations:** 1 Taekwondo Research Institute of Kukkiwon, Seoul Republic of Korea; 2 Department of physical education, Konkuk University, Seoul Republic of Korea

**Keywords:** Taekwondo, elementary students, physical fitness factors, meta-analysis, systematic review

## Abstract

**[Purpose]:**

We conducted a meta-analysis to evaluate the effects of Taekwondo training on the physical fitness factors in Korean elementary students comprehensively and quantitatively.

**[Methods]:**

We classified research studies published until November 2018 according to the Preferred Reporting Items for Systematic Reviews and Meta-Analyses guidelines and selected a total of 17 research items; a meta-analysis of these items was then conducted. We used the Comprehensive Meta-Analysis 3.0 and Review Manager 5.3 to analyze the mean effect size, study quality, and publication bias.

**[Results]:**

Taekwondo training improved the cardiopulmonary endurance, muscle endurance, and power of the elementary students, but was not practical or less useful on other physical fitness factors. The meta-regression analysis of the cardiopulmonary endurance and power items showed that the effect size was large when the sample size was small. Therefore, it is necessary to consider the sample size in interpreting the effect size for these two items. Further, during correction of the publication bias for the power items, the improvement effect by Taekwondo training was eliminated.

**[Conclusion]:**

Taekwondo training is helpful for improving the cardiopulmonary endurance and muscle endurance of Korean elementary students but is not useful for other physical fitness factors.

## INTRODUCTION

Taekwondo is a unique martial art and sport of Korea, which has gained international popularity as an Olympic sport since 2000.

Many individuals from approximately 209 countries are training in Taekwondo^1^, and more than 10.2 million individuals have a Taekwondo black belt^2^. Taekwondo training is performed in Taekwondo gymnasiums in each country. Therefore, the training program in Taekwondo gymnasiums has a significant factor on the effect of Taekwondo training. In Korea, 9,779 Taekwondo gymnasiums are operational as of November 2018^2^. Notably, most Taekwondo practitioners in Korea are elementary students^3^. Therefore, most Taekwondo gymnasiums in Korea operate as a training program for elementary students^4^.

For this reason, many studies have investigated the physiological^5^^,^^6^, psychological^7^^–^^9^, and sociological^10^^–^^12^ effects of Taekwondo on elementary students in Korea. In particular, we became interested in the physiological effects of Taekwondo on elementary students. As many of them practice Taekwondo, we think that Taekwondo will have a significant impact on the physical development of elementary students. Previous studies have reported the effects of Taekwondo training on children’s physical fitness^5^^,^^6^^,^^13^ and obesity^14^^–^^16^. However, the effects of Taekwondo training on the same physical fitness factors greatly varied among studies. Therefore, a systematic review^17^ and a meta-analysis^18^ were conducted to determine the effectiveness of Taekwondo training in children. However, they lack a publication bias analysis and a quality analysis for individual studies. Thus, the validity of these studies is low. Conversely, a meta-analysis conducted overseas investigated injury in relation to Taekwondo^19^. However, searching for meta-analyses conducted overseas, which investigated the effects of Taekwondo training in children, is difficult.

Therefore, we performed a systematic review and meta-analysis to determine the effects of Taekwondo training on the physical fitness factors in Korean elementary students.

## METHODS

This study was approved by the local ethics committee (7001355-201804-E-077) of our institution and was conducted in accordance with the Declaration of Helsinki principles. The systematic review process was based on the Preferred Reporting Items for Systematic Reviews and Meta-Analyses (PRISMA) statement^20^ and the Cochrane Handbook for Systematic Reviews of Interventions^21^.

### Eligibility criteria

We searched for relevant studies from online databases and libraries in Korea and conducted a systematic review of the collected studies. We applied the inclusion criteria according to the pre-set Participants, Intervention, Comparisons, Outcomes, Study design (PICOS) conditions (Table 1)^21^.

**Table 1:** Inclusion criteria according to the PICOS conditions

Items	Detailed inclusion criteria
Participants	Only studies involving healthy elementary school students were included. (Studies that included athletes, individuals with obesity, and patients as subjects were excluded.)
Intervention	Only studies that applied Taekwondo training for a specified period were included. (Studies combining Taekwondo with other treatments were excluded.)
Comparison	Only studies with control groups were included.
Outcomes	Only studies describing the means, standard deviations, and sample sizes for the physical fitness factors were included.
Study designs	Non-randomized controlled trials were included. (There are very few randomized controlled trials related to this topic in Korea.) (Studies with unclear training periods were excluded.)

### Data searches and sources

Studies published until November 2018 were searched using the Research Information Sharing Service (RISS), Korean Studies Information Service System (KISS), DBPIA, and Newnonmun. We used the following search keywords: “elementary AND Taekwondo AND physical fitness” and “child AND Taekwondo AND physical fitness.” Three investigators independently retrieved the data and compared the results.

### Data extraction

For the final 17 selected single studies^5^^,^^15^^,^^22^^–^^36^, three investigators independently extracted data according to the PICOS conditions. Further, we extracted data on the means, standard deviations, and sample sizes from each study.

### Assessment of risk of bias

Three investigators independently assessed the risk of bias from all single studies using the Risk of Bias Assessment tool for Non-randomized Studies (RoBANS)^37^. When the assessment results were similar among the three investigators, we used these results; in case of varying results, we used the results reaching consensus after a discussion. For the publication bias analysis^38^, we applied funnel plots^39^, the Egger’s test^40^, and the trim-and-fill test^41^.

### Data synthesis and statistical analysis

#### Analysis of the study characteristics

For the final 17 selected single studies, we entered the following data into the Excel 2016 program (Microsoft, WA, USA): publication year, publication type, study design, grades of students, sample size, training duration, exercise frequency per week, exercise time per session, exercise intensity, and training contents.

#### Meta-analysis and meta-regression analysis

We calculated the effect sizes for the post-training data of the experimental and control groups in each study; the selected studies presented only means and standard deviations and did not present raw data. Further, although the selected studies were non-randomized controlled trials, the pre-training values were similar between the groups by applying an equal division. Thus, we extracted the post-training data (means, standard deviations, and sample sizes) from each study and used them to calculate the effect size, weight, and 95% confidence intervals (CIs).

We calculated the effect size as Hedges’s g, not as Cohen’s d, as the latter may be overestimated when the sample size is small^42^, and most selected single studies had small sample sizes. The weight of the effect size was calculated as the inverse of the variance. We applied the fixed-effect model to calculate the mean effect sizes, as the subjects, measuring methods, and training methods were similar among the included studies. Therefore, we assumed that the effect sizes of the population of all included studies were similar^39^. Moreover, we calculated the effect sizes of the studies when there were three or more studies with the same outcome variables.

For the heterogeneity analysis of the calculated effect sizes, we applied the chi-square test for Cochran’s Q and used Higgin’s *I*^2^. We judged that the heterogeneity between the effect sizes was substantial when the p-value obtained using the chi-square test was less than .1037 and Higgin’s *I*^2^ was more than 50%^21^^,^^43^.

For additional explanation of the effect size heterogeneity, we selected the covariate and performed a meta-regression analysis using this covariate.

All statistical analyses were performed using the Comprehensive Meta-Analysis 3.0 (Biostat, NJ, USA) and Review Manager 5.3 (Cochrane Collaboration, Oxford, UK).

## RESULTS

### Selection and inclusion of studies

We collected 164 studies through a database search in the RISS, KISS, DBpia, and Newnonmun and additionally selected two Kukkiwon reports. Thus, the total number of collected studies was 166. Next, we excluded 63 duplicate studies (duplicate search or publication of the thesis into a journal).

We reviewed the research titles and abstracts of the 103 remaining selected studies and further excluded 25 studies that included individuals with obesity, patients, and athletes as their subjects. This was performed to maintain the homogeneity of the subjects. Moreover, we excluded five studies that combined additional treatments besides a general training program (warm-up, physical training, taekwondo training, and cool-down) in a Taekwondo gymnasium. This was conducted to objectify the effect of Taekwondo training. Additionally, we excluded three studies that did not explain the training duration and five studies with unsearchable full text. Therefore, we selected 65 studies after excluding 38 studies.

**Figure 1:**
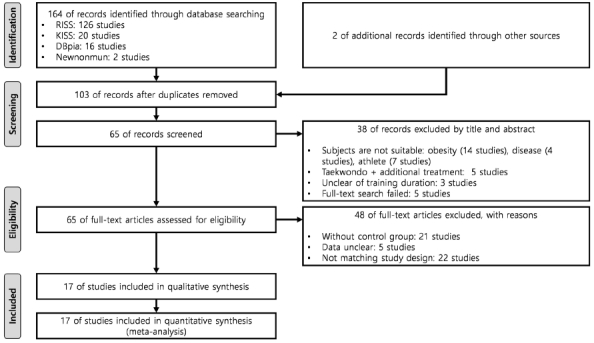
Preferred Reporting Items for Systematic Reviews and Meta-Analyses flow diagram

We reviewed the full text of the 65 selected studies and classified them according to the PICOS criteria. Thereafter, we excluded 21 studies with no control group, five studies with unclear statistics, and 22 studies with an uncertain training duration. Therefore, we finally included 17 studies after excluding 48 studies. We described the data selection process in a PRISMA guideline^20^ flow diagram (Figure 1).

### Selection of outcome variables

Sit-up and hand-grip strengths were presented in 16 studies. Standing broad jump was presented in 12 studies and sit and reach in 10 studies. A single-leg stance with the eyes closed was presented in eight studies and trunk forward flexion in five studies. Other physical fitness factors were presented in less than four studies (Table 2).

**Table 2:** Summary of the outcome variables

No	Author	Year	Detailed inclusion criteria
1	Chae	2002	200-m run, sit-up (maximum), back muscle strength, trunk forward flexion, single-leg stance with the eyes closed, standing broad jump
2	Chang	2014	Left grip strength, right grip strength, sit-up (60 seconds), standing broad jump, 20-m multistage shuttle run, sit and reach
3	Choo	2011	sit-up (maximum), 200-m run, standing broad jump, side-step (20 seconds), trunk forward flexion, single-leg stance with the eyes closed
4	Jeong & Kim	2001	left grip strength, right grip strength, sit-up (60 seconds), shuttle run (agility), standing broad jump, 800-m run, trunk forward flexion, single-leg stance with the eyes closed
5	Jung & Lee	2016	grip strength, sit-up (60 seconds), standing broad jump, 50-m run, sit and reach, 1600-m run
6	Kim	2005	sit-up (60 seconds), single-leg stance with the eyes closed, jump rope (60 seconds)
7	Kim	2012a	20-m multistage shuttle run, sit and reach, sit-up (60 seconds), grip strength, standing broad jump, single-leg stance with the eyes closed
8	Kim	2012b	200-m running, sit-up (60 seconds), standing broad jump, sit and reach, side-step (20 seconds), single-leg stance
9	Kim	2014	push-up (30 seconds), sit-up (60 seconds), standing broad jump, 10-m shuttle run (agility), jump rope (60 seconds), sit and reach
10	Koh	2018	left grip strength, right grip strength, sit and reach, standing broad jump, sit-up (maximum), 15-m shuttle run (endurance)
11	Lee	2014	grip strength, sit-up (60 seconds), trunk forward flexion, vertical jump, single-leg stance with the eyes closed
12	Lee & Choi	2014	grip strength, sit-up (30 seconds), 20-m multistage shuttle run, standing broad jump
13	Ma	2010	grip strength, sit-up (30 seconds), motor activity, vertical jump, sit and reach, single-leg stance with the eyes closed
14	Moon & Kim	2007	maximal oxygen uptake, back-muscle strength, left grip strength, right grip strength, sit-up (60 seconds), trunk forward flexion, 20-m shuttle run (agility), throwing a handball (power)
15	Oh & Park	2016	back-muscle strength, sit and reach, side-step (60 seconds), vertical jump
16	Song	2016	grip strength, standing broad jump, sit-up (60 seconds), side-step (60 seconds), sit and reach
17	Yang	2015	right grip strength, left grip strength, sit-up (60 seconds), Harvard step, sit and reach, standing broad jump

### Study, subject, and intervention characteristics

We summarized the characteristics of the selected studies in Table 3. The experimental design of all included studies was a non-randomized controlled trial. The total sample size of the experimental groups was 250, and that of the control groups was 248. However, the sample sizes of 14 studies ranged from 10 to 20. Thus, most of the selected studies had small sample sizes of 10‒20.

**Table 3:** Summary of the study characteristics (17 included studies)

No	Author (year)	Publication type	Study design	Subjects						Interventions			
				Grades	Exp (y)	Cont (y)	Exp (N)	Cont (N)	Duration (wk)	Frequency (sessions/wk)	Time (min/ session)	Intensity	Training contents
1	Chae (2002)	Thesis	NRCT	2	ND	ND	25	25	12	5	60	50%‒70% of the HRmax	Warm-up (stretching); Physical training (shuttle run, one-leg jump, and jump over); Basic movement (kicking); Poomsae training; Cool-down (stretching)
2	Chang (2014)	Thesis	NRCT	3‒6	11.7	11.5	15	15	12	4	60	ND	Warm-up (gymnastics, stretching); Physical training (burpee, sit and jump, and PT jump); Basic movement (standing, kicking, blocking, and punching); Poomsae training; Sparring; Cool-down (gymnastics, stretching)
3	Choo (2011)	Thesis	NRCT	2‒6	11.6	11.0	10	10	8	ND	60	1‒2 wk: 50%‒60% of the HRmax 3‒5 wk: 60%‒70% of the HRmax 6‒8 wk: 70%‒80% of the HRmax	Warm-up (stretching); Physical training (rope jump and interval training); Basic movement (standing, basic kicking, quick kicking, and cross kicking); Poomsae training (basic Poomsae and music Poomsae); Sparring; Self-defense; Cool-down (stretching)
4	Jeong & Kim (2001)	Journal	NRCT	5‒6	11.8	12.0	30	30	12	5	60	ND	Warm-up (stretching); Physical training (burpee, sit and jump, and butterfly jump); Basic movement (standing, kicking, blocking, and punching); Poomsae training; Sparring; Cool-down (stretching, gymnastics)
5	Jung & Lee (2016)	Thesis	NRCT	ND	12.4	12.6	10	10	12	3	60	1‒4 wk: 40%‒50% of the HRmax 5‒8 wk: 50%‒60% of the HRmax 9‒12 wk: 60%‒70% of the HRmax	Warm-up (stretching); Physical training (PT jump, sit and jump, burpee, knee-pull jump, sit-up, vertical jump, push-up, and mountain climber); Basic movement (standing, blocking, punching, basic kicking, meet kicking, and change stepping); Sparring; Cool-down (stretching)
6	Kim (2005)	Thesis	NRCT	ND	11.7	11.9	15	15	12	5	60	60%‒70% of the HRmax	Warm-up (gymnastics, Taekwon-robic); Physical training (sit-up, push-up, isometric training, standing broad jump, jump and spinning, vertical jump, jump over, rope jump, zigzag run, mountain climber, shuttle run, long-run, gymnastics, single-leg stance with the eyes closed, PT jump, burpee, running backwards, and diamond step); Basic movement (standing, blocking, punching, kicking, and breaking); Poomsae training; Sparring
7	Kim (2012a)	Thesis	NRCT	3‒6	12.1	13.1	12	10	12	4	60	15 RPE	Warm-up (running, gymnastics, stretching, and flexibility exercise); Basic movement (standing, basic kicking, punching, and blocking); Poomsae training; Sparring; Cool-down
8	Kim (2012b)	Thesis	NRCT	2‒4	10.8	10.5	10	10	12	5	60	50%‒70% of the HRmax	Warm-up (stretching); Physical training (shuttle run, knee-pull jump, and one-leg jump); Basic movement (kicking, blocking, and punching); Cool-down (stretching)
9	Kim (2014)	Thesis	NRCT	3‒4	10.5	10.5	10	10	12	5	60	ND	Warm-up (stretching, Taekwon gymnastics); Physical training (10-m shuttle run, zigzag run, rope jump, side-step, and jumping exercise); Basic movement (standing, punching, kicking, blocking, and quick stepping); Poomsae training; Sparring; Self-defense; Cool-down (stretching and contemplation)
10	Koh (2018)	Thesis	NRCT	1‒4	9.3	9.6	12	11	12	5	50	ND	Warm-up (stretching); Physical training (push-up, sit-up, knee-pull jump, trunk forward flexion, bridge exercise, and lie down and bike riding); Basic movement (standing, basic kicking, moving forward kicking, meet kicking, compound kicking, and lie down and kicking); Poomsae training; Sparring; Cool-down (stretching)
11	Lee (2014)	Thesis	NRCT	3‒6	11.9	11.7	10	10	12	5	60	ND	Physical training (balance exercise, shuttle run, jump exercise, muscle endurance exercise); Basic movement (kicking, meet kicking, Taekwon gymnastics); Poomsae training; Self-defense; Sparring
12	Lee & Choi (2014)	Journal	NRCT	5‒6	12.5	12.3	11	11	24	5	60	ND	Warm-up (stretching, running); Physical training (jumping, push-up, burpee test, rope jump); Basic movement (kicking, standing, blocking); Poomsae training; Cool-down (stretching)
13	Ma (2010)	Thesis	NRCT	ND	11.4	11.4	10	10	12	5	60	ND	Warm-up (stretching); Physical training (running); Basic movement (kicking, blocking); Poomsae training; Sparring; Cool-down (stretching)
14	Moon & Kim (2007)	Journal	NRCT	ND	12.1	12.3	10	10	12	5	70	ND	Warm-up (Taekwon gymnastics, stretching); Physical training (10m shuttle run, push-up, sit-up, PT jump, vertical jump, one leg jump); Basic movement (standing, basic kicking, punching, blocking); Poomsae training; Sparring (1:1 sparring, group sparring); Cool-down (Taekwon gymnastics, stretching)
15	Oh & Park (2016)	Journal	NRCT	ND	13.1	13.0.	17	18	16	4	60	RPE (~11‒13)	Warm-up (stretching); Basic movement (standing, blocking, punching, kicking); Poomsae training; Cool-down (stretching)
16	Song (2016)	Thesis	NRCT	4‒6	ND	ND	30	30	12	5	60	ND	Warm-up (running, stretching); Physical training (rope jump and circuit training); Basic movement (basic kicking, compound kicking, blocking, and Taekwon gymnastics); Poomsae training; Sparring; Self-defense
17	Yang (2015)	Thesis	NRCT	5‒6	13.0	13.0	13	13	12	3	60	1‒3 wk: 50%‒60% of the HRR 4‒12 wk: 70% of the HRR	Warm-up (stretching); Physical training (running, push-up, sit-up, jump over, PT jump, vertical jump, rope jump, knee-pull jump, and circuit training); Basic movement (basic kicking, meet kicking, and quick kicking); Sparring; Cool-down (stretching)

Fourteen studies applied Taekwondo training for 12 weeks. Thus, most studies had 12 weeks of training duration. Eleven studies applied an exercise frequency of five sessions per week. Therefore, the most frequently applied exercise frequency was five sessions per week. Fifteen studies applied an exercise time of 60 minutes per session. Thus, the most frequently applied exercise time was 60 minutes per session. The average exercise intensity in one study was 55% of the maximum heart rate (HRmax); two studies, 60% of the HRmax; and two studies, 65% of the HRmax. Further, the average exercise intensity in one study was 60% of the heart rate reserve (HRR); one study, rate of perceived exertion (RPE) of 12; and one study, RPE of 15. Conversely, nine studies did not report the exercise intensity. However, we found that the exercise intensities presented in some studies were similar.

The training contents included warm-up, physical training, basic movement, Poomsae training, self-defense, and cool-down. For the warm-up phase, most of the studies (15 studies) used stretching. For the physical training phase, 34 various types of jumping exercises were used in a total of 13 studies; push-up was used in six studies; burpee and sit-up were both used in five studies. Two types of shuttle run were used in four studies; the balance exercise, circuit training, mountain climber, and zigzag run were each used in two studies. Moreover, the side-step exercise, bridge exercise, interval training, lie down and bike riding, muscle endurance exercise, trunk forward flexion, and diamond step exercise were all used in one study. Therefore, the frequency of the training contents used for physical training in the Korean Taekwondo gymnasiums was higher in the order of power exercise, muscle endurance exercise, and cardiopulmonary endurance exercise. However, training programs to improve muscle strength were rarely used. For the basic movement phase, 28 various types of the kicking movement were used in all studies, blocking movement in 12 studies, and standing movement in 11 studies. Further, the punching movement was used in nine studies and both the stepping movement and the Taekwon gymnastics in two studies.

Poomsae training was used in 14 studies, and the used Poomsae was Taegeuk Poomsae. Sparring was used in 14 studies and self-defense in four studies. For the cool-down phase, stretching was used in 13 studies.

### Risk of bias assessment

**Figure 2:**
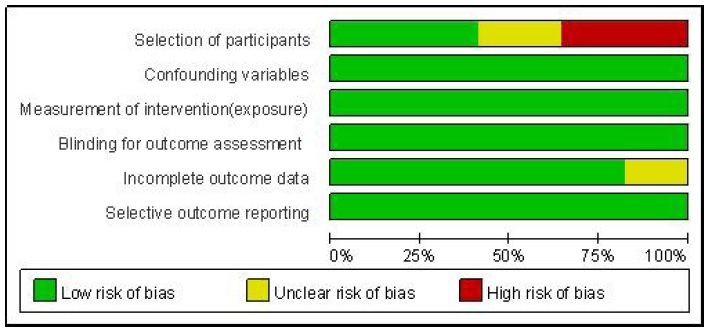
Risk of bias

For the selection of participants, 35.3% (six studies) had a high risk of bias; 23.5% (four studies) had an unclear risk of bias; and 41.2% (seven studies) had a low risk of bias. For incomplete outcome data, 17.6% (three studies) had an unclear risk of bias, and 83.4% (14 studies) had a low risk of bias. For confounding variables, measurement of intervention, blinding for outcome assessment, and selective outcome reporting, all studies had a low risk of bias (Figure 2).

### Effects of Taekwondo training

#### Effect of Taekwondo training on muscle strength

**Figure 3:**
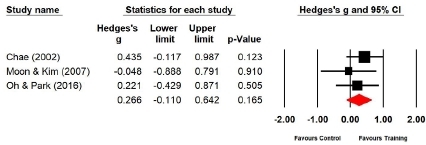
Effect size of Taekwondo training for back muscle strength CI, confidence interval

Three studies (n=105 subjects) reported the back muscle strength. After Taekwondo training, the back muscle strength of the experimental group increased by 8.9% compared with that of the control group; the difference was not significant (Hedges’s g=0.226; 95% CI=-0.110‒0.642). Therefore, Taekwondo training had no effect on the back muscle strength (Figure 3). Further, the effect size for the back muscle strength showed a low heterogeneity (*I*^2^ =0.000%; Q=0.915, p=.633).

**Figure 4:**
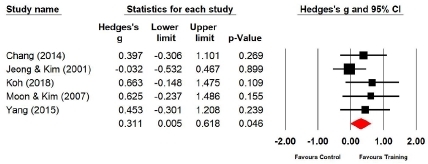
Effect size of Taekwondo training for right grip strength CI, confidence interval

Four studies (n=159 subjects) reported the right grip strength. After Taekwondo training, the right grip strength of the experimental group increased by 12.2% compared with that of the control group, and the difference was significant (Hedges’s g=0.311; 95% CI=0.005‒0.618). Therefore, the effect size of Taekwondo training on the right grip strength was small (Figure 4). Further, the effect size for the right grip strength showed a low heterogeneity (*I*^2^ =0.000%; Q=3.243, p=.518).

**Figure 5:**
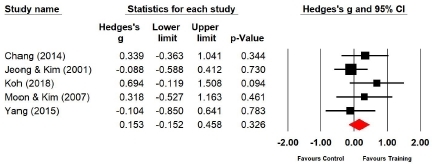
Effect size of Taekwondo training for left grip strength CI, confidence interval

Five studies (n=159 subjects) reported the left grip strength. After Taekwondo training, the left grip strength of the experimental group increased by 6.1% compared with that of the control group, and the difference was not significant (Hedges’s g=0.153; 95% CI=-0.152‒0.458). Therefore, Taekwondo training had no effect on the left grip strength (Figure 5). Moreover, the effect size for the left grip strength showed a low heterogeneity (*I*^2^ =0.000%; Q=3.497, p=.478).

#### Effect of Taekwondo training on muscle endurance

**Figure 6:**
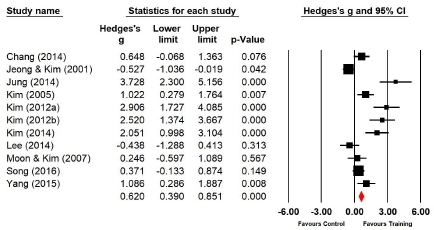
Effect size of Taekwondo training for muscle endurance during sit-up CI, confidence interval

Eleven studies (n=328 subjects) applied sit-ups. During sit-ups, the muscle endurance of the experimental group increased by 23.2% compared with that of the control group, and the difference was significant (Hedges’s g=0.620; 95% CI=0.390‒0.851). Therefore, the effect size of Taekwondo training on muscle endurance during sit-ups was medium (Figure 6). Further, the effect size showed a high heterogeneity (*I*^2^ =87.591%; Q=80.584, p=.000).

#### Effect of Taekwondo training on power

**Figure 7:**
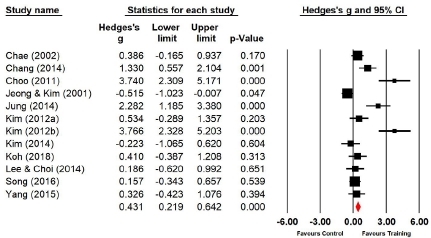
Effect size of Taekwondo training for power during standing broad jump CI, confidence interval

Twelve studies (n=373 subjects) applied standing broad jump. During standing broad jumps, the power of the experimental group increased by 16.7% compared with that of the control group, and the difference was significant (Hedges’s g=0.431; 95% CI=0.219‒0.642). Therefore, the effect size of Taekwondo training on power during standing broad jumps was below medium (Figure 7). Moreover, the effect size showed a high heterogeneity (*I*^2^ =85.346%; Q=75.064, p=.000).

**Figure 8:**
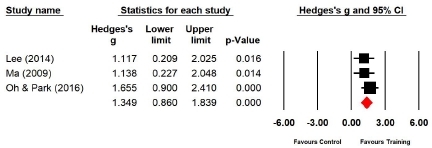
Effect size of Taekwondo training for power during vertical jump CI, confidence interval

Three studies (n=75 subjects) applied vertical jump. During vertical jumps, the power of the experimental group increased by 41.1% compared with that of the control group, and the difference was significant (Hedges’s g=1.349; 95% CI=0.860‒1.839). Therefore, the effect size of Taekwondo training on power during vertical jumps was very large (Figure 8). Further, the effect size showed a low heterogeneity (*I*^2^ =0.000%; Q=0.981, p=.612).

#### Effect of Taekwondo training on cardiopulmonary endurance

**Figure 9:**
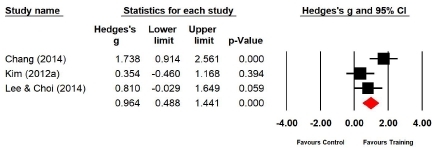
Effect size of Taekwondo training for cardiopulmonary endurance during the 20-m multistage shuttle run CI, confidence interval

Three studies (n=74 subjects) applied the 20-m multistage shuttle run. During the 20-m multistage shuttle run, the cardiopulmonary endurance of the experimental group increased by 33.2% compared with that of the control group, and the difference was significant (Hedges’s g=0.964; 95% CI=0.488‒1.441). Therefore, the effect size of Taekwondo training on cardiopulmonary endurance during the 20-m multistage shuttle run was large (Figure 9). Further, the effect size showed a high heterogeneity (*I*^2^ =64.159%; Q=5.580, p=.061).

**Figure 10:**
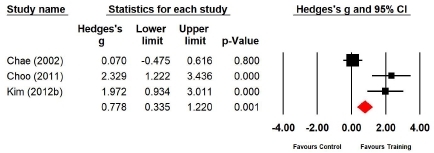
Effect size of Taekwondo training for cardiopulmonary endurance during the 200-m run CI, confidence interval

Three studies (n=90 subjects) applied the 200-m run. During the 200-m run, the cardiopulmonary endurance of the experimental group increased by 28.2% compared with that of the control group, and the difference was significant (Hedges’s g=0.778; 95% CI=0.335‒1.220). Therefore, the effect size of Taekwondo training on cardiopulmonary endurance during the 200-m run was medium (Figure 10). Further, the effect size showed a high heterogeneity (*I*^2^ =89.723%; Q=19.460, p=.000).

#### Effect of Taekwondo training on flexibility

**Figure 11:**
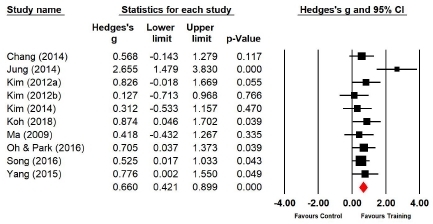
Effect size of Taekwondo training for flexibility during the sit and reach CI, confidence interval

Ten studies (n=276 subjects) applied the sit and reach. During the sit and reach, the flexibility of the experimental group increased by 24.5% compared with that of the control group, and the difference was significant (Hedges’s g=0.660; 95% CI=0.421‒0.899). Therefore, the effect size of Taekwondo training on flexibility during the sit and reach was above medium (Figure 11). Moreover, the effect size showed a low heterogeneity (*I*^2^ =37.834%; Q=14.477, p=.106).

**Figure 12:**
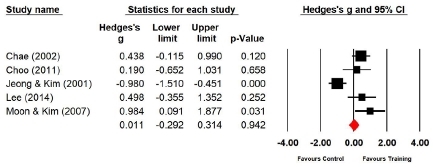
Effect size of Taekwondo training for flexibility during trunk forward flexion CI, confidence interval

Five studies (n=170 subjects) applied trunk forward flexion. During trunk forward flexion, the flexibility of the experimental group increased by 0.4% compared with that of the control group, and the difference was not significant (Hedges’s g=0.011; 95% CI=-0.292‒0.314). Therefore, Taekwondo training had no effect on flexibility during trunk forward flexion (Figure 12). Further, the effect size for trunk forward flexion showed a high heterogeneity (*I*^2^ =81.614%; Q=21.755, p=.000).

#### Effect of Taekwondo training on balance ability

**Figure 13:**
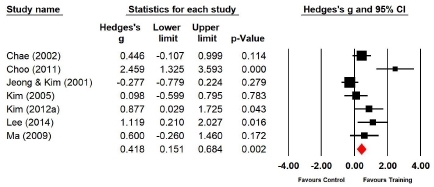
Effect size of Taekwondo training for the balance ability during the single-leg stance with the eyes closed CI, confidence interval

Seven studies (n=222 subjects) applied the single-leg stance with the eyes closed. During the single-leg stance with the eyes closed, the balance ability of the experimental group increased by 16.2% compared with that of the control group, and the difference was significant (Hedges’s g=0.418; 95% CI=0.151‒0.684). Therefore, the effect size of Taekwondo training on balance ability during the single-leg stance with the eyes closed was below medium (Figure 13). Moreover, the effect size showed a high heterogeneity (*I*^2^ =75.521%; Q=24.511, p=.000).

### Meta-regression analysis

The meta-regression analysis was performed only for the sit-up and standing broad jump, as the effect sizes of the two variables were significant and had considerable heterogeneity, and there were a sufficient number of studies (≥10)^44^.

**Figure 14:**
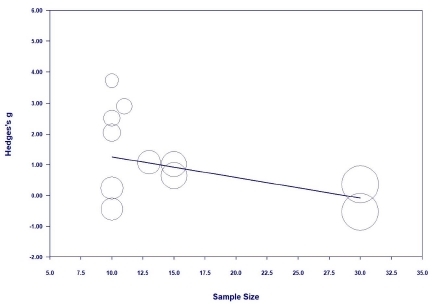
Meta-regression analysis for the sit-up

**Figure 15:**
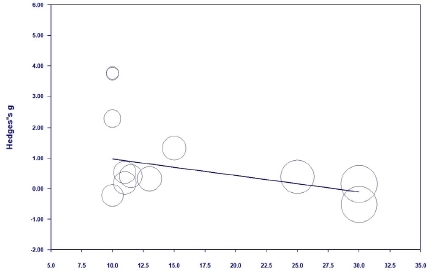
Meta-regression analysis for the standing broad jump

For the sit-up, as the sample size increased, the effect size decreased; this result was significant (Z=-2.11, p=.035) (Figure 14). For the standing broad jump, as the sample size increased, the effect size decreased; this result was significant (Z=-4.34, p=.000) (Figure 15).

### Publication bias analysis

**Figure 16:**
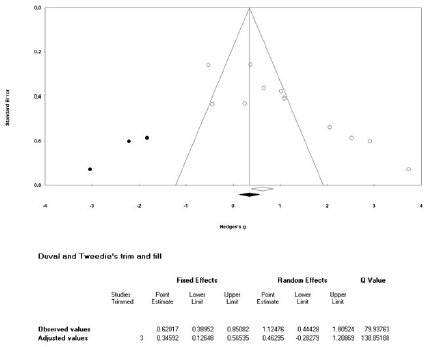
Publication bias analysis for the sit-up

The publication bias analysis was performed only for the variables assessed in more than ten studies in the calculation of the mean effect sizes (sit-up and standing broad jump). Based on the funnel plot for the sit-up^39^, the effect sizes were asymmetrically distributed (Figure 16). Thus, we conducted an asymmetric test using Egger’s regression test^40^; a publication bias was found for the sit-up (t=4.318, df=9, p=.002). Therefore, we performed the trim-and-fill test^41^ to correct the mean effect size. The corrected mean effect size was calculated as 0.346, which was 44.2%p lower than the mean effect size before correction (0.620). Further, as the 95% CI of the corrected mean effect size was 0.126‒0.565, it was still significant. Therefore, although a publication bias was found in the meta-analysis for the sit-up, it was confirmed that the bias was not severe enough to change the outcome.

**Figure 17:**
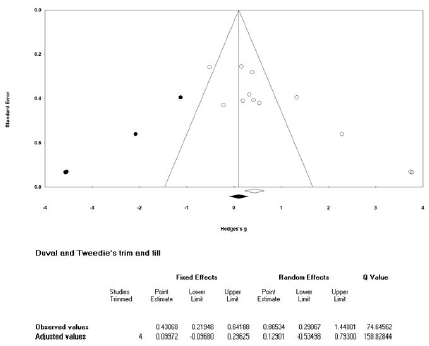
Publication bias analysis for the standing broad jump

Based on the funnel plot for the standing broad jump, the effect sizes were asymmetrically distributed (Figure 17). Thus, we also conducted the asymmetric test using Egger’s regression test; a publication bias was also found for the standing broad jump (t=4.662, df=10, p=.001). Therefore, we performed the trim-and-fill test to correct the mean effect size. The corrected mean effect size was calculated as 0.100, which was 76.8%p lower than the mean effect size before correction (0.431). Moreover, the 95% CI of the mean effect size changed from 0.219‒0.642 to -0.097‒0.296, which was not significant. Therefore, the meta-analysis of the standing broad jump revealed a publication bias, which was confirmed to be severe enough to change the outcome.

## DISCUSSION

We selected only studies with similar subjects and training types to improve the validity and objectivity of the meta-analysis results.

### Characteristics of the selected studies

Most of the studies included herein did not randomly divide their experimental and control groups. The fact that a non-randomized control group was used has a negative impact on the validity of this study. However, although the studies included had a non-randomized controlled trial design, both the experimental and control groups were recruited from the same population for the same duration; further, the initial characteristics of the subjects, such as residential area, grade, and age, were similar. In fact, as the duration and institution of recruitment were the same, the risk of bias for participant selection was low^37^. Thus, we evaluated that the validity of this study was not considerably undermined.

Most of the selected studies had small sample sizes. If the sample size is small, the effect size may be extreme^37^. In fact, the meta-regression analysis of this study showed that the effect size was large when the sample size was small. Therefore, it is necessary to consider the small sample size when interpreting the mean effect size in this study.

In most studies, the Taekwondo training period was 12 weeks, and the exercise frequency was five sessions per week; further, the exercise time per session was 60 minutes. Therefore, the mean effect sizes in this study can be regarded as the result of the same training period, training frequency, and exercise time per session.

The exercise intensity is an essential factor in exercise prescription, which determines exercise effects, along with frequency, time, type, volume, and progression^45^. In this study, nine out of the 17 studies did not report the exercise intensity. Thus, we could not obtain information on the exercise volume. However, we found that the exercise intensities presented in the eight remaining studies were similar. We also attempted to collect similar studies through a systematic review. Therefore, we judged that the exercise intensity of the nine studies that did not report the exercise intensity would be similar to that of the eight studies.

In this study, stretching was used during warm-up and cool-down. The main exercise program consisted of Taekwondo training and physical training. We think that this main exercise program has a significant impact on physical fitness factors.

### Risk of bias assessment

Six studies recruited students who have already started Taekwondo training and classified them into the experimental group and control group. Thus, we judged the risk of bias to be high for the selection of participants in these studies. In four studies, the experimental group consisted of students who had just started Taekwondo training, and the control group consisted of students from nearby elementary schools. Therefore, the subjects in these four studies were not recruited from the same institution. However, the recruitment area of the subjects was the same, and the initial characteristics (i.e., grade and age) between the experimental group and the control group were similar. Therefore, we judged the risk of bias for the selection of participants to be unclear in these studies. The seven remaining studies organized the experimental group and the control group under the same conditions. Thus, we judged the risk of bias for the selection of participants to be low in these studies.

Three studies did not report dropout of the subjects. Therefore, we did not know whether there was any dropout. Thus, we judged the risk of bias for incomplete outcome data to be unclear in these studies.

For the confounding variables, measurement of intervention, blinding for outcome assessment, and selective outcome reporting, all studies were judged to have a low risk of bias.

To summarize the results of the assessment of the risk of bias, there is an issue on the risk of bias for the selection of participants. However, both the experimental group and the control group consisted of students recruited from the same area with the same age and physique, and the risk of bias was not high in most evaluation areas. Therefore, we concluded that the risk of bias in this study does not negatively affect the study results.

### Effect size and heterogeneity

Taekwondo training did not improve the back muscle strength and left grip strength; there was only a minimal improvement in the right grip strength. Thus, we analyzed the Taekwondo training program to determine the cause; in all studies included in the analysis, exercise programs for improving muscle strength were rarely used. Generally, to improve muscle strength, a high-intensity resistance exercise repeated for 8‒12 times per set was recommended^46^. Further, a high-intensity resistance exercise requires a weight machine or a tool. However, most studies included in the analysis did not have a high-intensity resistance exercise program. It was unclear why a high-intensity resistance exercise was excluded from the training program in Taekwondo gymnasiums in Korea. However, we think that this may be attributed to the fact that Taekwondo is an exercise that uses the body without special tools. The critical point is that a high-intensity resistance exercise must be included to improve muscle strength during training in Taekwondo gymnasiums.

Taekwondo training had a greater than medium effect on muscle endurance during sit-ups. We analyzed the training program to determine the cause of the improved muscle endurance; the Taekwondo training program included various exercises that could affect muscle endurance enhancement, such as various kicking motions, sit and jump, burpee, knee-pull jump, push-up, sit-up, mountain climber, bridge exercise, and lie down and bike riding. Park^47^ analyzed electromyography findings according to the angle of the trunk or knee during a sit-up; when the angle of the trunk was 90°, the angle of the knee was smaller, and the development of the rectus abdominis and obliquus externus was better. The kicking motion is a motion in which the angle of the knee is small, and the angle of the trunk is close to 90°. Therefore, we judged that the repetitive kicking motions during Taekwondo training might have improved the muscle endurance during sit-ups. We also determined that the various core exercises included in the Taekwondo training program helped improve the muscle endurance.

Taekwondo training had a below medium effect on power during standing broad jumps and had a very large effect on power during vertical jumps. We analyzed the training program to determine the cause of the improved power; in all studies included in the analysis, various types of jumping exercise were used.

Most of the jumping exercises were vertical jumps, such as rope jump, one-leg jump, sit and jump, knee-pull jump, jump over, and butterfly jump. We think that the power dramatically improved because of the abovementioned reasons. In general, the rectus femoris (25.95%), vastii (20.57%), gluteus maximus (16.09%), and soleus (14.4%) have the most significant impact on the vertical jumping ability^48^. Thus, repetitive jumping exercises help improve the jumping ability by strengthening the muscles listed above^49^. However, careful interpretation of the findings is needed because the number of the studies included in the analysis of the vertical jumps was small.

Taekwondo training had a large effect on the cardiopulmonary endurance during the 20-m multistage shuttle run and had more than a medium effect during the 200-m run. We analyzed the training program to determine the cause of the cardiopulmonary endurance improvement; there was no shuttle run exercise in the training program of all selected studies for the analysis of the 20-m multistage shuttle run. Further, there was no running exercise in the training program of all selected studies for the analysis of the 200-m run. Despite such, the cardiopulmonary endurance might have improved because of the effect of the medium to high intensity and long-term activity during the training period. Generally, to improve adult cardiopulmonary endurance, exercise types that use large muscle groups, such as running, biking, swimming, and cross-country skiing, are recommended. Further, the recommended exercise intensity is 50%‒85% of the maximal exercise capacity; the recommended exercise frequency is 3‒5 times per week; and the recommended exercise time is 20‒60 minutes per session^50^. If proper physical training is conducted, the effect of endurance training in adults can also be observed in children^51^. In all selected studies herein, the children performed physical training and Taekwondo training for 50‒60 minutes at an exercise intensity of 50%‒80% of the HRmax each time during the training period (12 weeks). Therefore, we determined that such long-term training improves the cardiopulmonary endurance of children. According to previous studies^52^^-^^54^, individuals with lower cardiopulmonary endurance have a more significant improvement in cardiopulmonary endurance than those with higher cardiopulmonary endurance. This result supports our findings.

Taekwondo training had more than a medium effect on flexibility during the sit and reach and did not enhance such during trunk forward flexion. We analyzed the training program to determine the cause of the flexibility improvement; stretching was most commonly used during warm-up and cool-down. Conversely, only a few flexibility exercises were included in the physical training program. However, during Taekwondo training, the basic movement and the Poomsae program included several motions related to the extension of the joints. The flexibility of the children increased because of the frequent exercise related to flexibility improvement in the warm-up, cool-down, and main exercise phases. Unlike that in the sit and reach, it is difficult to explain precisely why the flexibility during trunk forward flexion did not improve. However, based on our long clinical experience, it may be difficult to measure the flexibility during trunk forward flexion accurately when the subject is afraid of developing any injury when bending the upper body in the forward posture.

Taekwondo training had a below medium effect on the balance ability during the single-leg stance with the eyes closed. The balance ability might have improved through Taekwondo training because various redirections, moving actions, and changes between attacks and defenses are repeated in basic movements, sparring, kicking, and Poomsae^55^.

### Meta-regression analysis

In the meta-regression analysis, we determined the sample size as an independent variable. In both sit-up and standing broad jump, when the sample size was large, the effect size was smaller; this result was significant. In conclusion, we confirmed that the cause of heterogeneity for the sit-up and standing broad jump was the sample size. Therefore, when interpreting the effects of Taekwondo training, the effect size may be overestimated if the sample size is small.

### Publication bias analysis

The mean effect size for the sit-up was found to have a publication bias. In the trim-and-fill test^41^, the modified mean effect size decreased by 44.2%p. However, the adjusted mean effect size was also significant. Therefore, the publication bias for the mean effect size for the sit-up was not severe enough to change the conclusion. The mean effect size for the standing broad jump was also found to have a publication bias. In the trim-and-fill test^41^, the modified mean effect size decreased by 76.8%p; the adjusted mean effect size was not significant. Therefore, the publication bias for the mean effect size for the standing broad jump was severe enough to change the conclusion.

## CONCLUSIONS

We conducted a systematic review and meta-analysis on the effects of Taekwondo training on the physical fitness factors in Korean elementary students. Based on the results of the analysis, we made the following conclusions considering both the heterogeneity of the effect size and the publication bias.

Taekwondo training in elementary students in Korea is helpful in improving muscle endurance and cardiopulmonary endurance but does not help improve muscle strength and balance. Further, the sample size is the control variable for the effect size. If the sample size is small, the effect of Taekwondo training can be overestimated.
